# Optimization of a protocol for the evaluation of antibody responses to human papillomavirus (HPV) vaccination in low-resource settings

**DOI:** 10.1186/s12905-022-01821-y

**Published:** 2022-06-16

**Authors:** Emmanuel Timmy Donkoh, Edward Tieru Dassah, Ellis Owusu-Dabo

**Affiliations:** 1 Center for Research in Applied Biology, University of Energy and Natural Resources, Sunyani, Ghana; 2 Department of Population, Family and Reproductive Health, School of Public Health, Kwame Nkrumah University of Science and Technology, Kumasi, Ghana; 3 Department of Global and International Health, School of Public Health, Kwame Nkrumah University of Science and Technology, Kumasi, Ghana

**Keywords:** HPV, Quadrivalent vaccine, Antibodies, Immunogenicity, Ghana

## Abstract

**Introduction:**

Available human papillomavirus (HPV) vaccines could have an important primary role in cervical cancer prevention once their long-term immunogenicity and safety are evaluated at the population level. The aim of this study was to optimize an assay to be used in evaluating the long-term durability of HPV vaccine response following a pilot vaccination of adolescent girls in Ghana.

**Methods:**

A rapid, high-throughput, indirect enzyme-linked immunosorbent assay (ELISA) was optimized for the detection and quantitation of anti-HPV L1 (late expression protein: types 6, 11, 16 and 18) immunoglobulin G (IgG) in human serum (*n* = 89). The utility of the assay was demonstrated using serum collected from a cohort of pre-adolescent girls (n = 49) previously vaccinated with a quadrivalent vaccine and non-immune serum obtained from age-matched controls (n = 40).

**Results:**

The assay showed good discrimination of antibody levels between cases and control sera: seroprevalence of anti-HPV IgG antibodies was significantly higher among vaccinated than unvaccinated girls for both HPV-16 (63.3% vs. 12.5%; p < 0.001) and HPV-18 (34.7% vs. 20.0%; p = 0.042), respectively. Thirty-six months after receiving the third dose of vaccine, significantly higher mean anti-HPV-16 (0.618 vs. 0.145), anti-HPV-18 (0.323 vs. 0.309), and anti-HPV-6 (1.371 vs. 0.981) antibody levels were measured, compared to unvaccinated girls (all p < 0.05). A correlation between optical density and antibody activity indicated assay sensitivity to increasing levels of antibody activity.

**Conclusion:**

We have successfully optimized and implemented a robust and sensitive assay for the evaluation of antibody responses among immunized adolescent girls for monitoring future large-scale HPV vaccination studies in low-income settings. Our results demonstrated greater immunoglobulin G antibody activity within serum drawn from adolescent girls immunized 36 months prior.

**Supplementary Information:**

The online version contains supplementary material available at 10.1186/s12905-022-01821-y.

## Background

Cervical cancer is a leading cause of female cancer-related morbidity and mortality in Ghana [1]. Over 3000 women are diagnosed with, and more than 2000 women die from the disease each year in Ghana [2]. A vast majority of cervical cancers are attributable to persistent infection with oncogenic genotypes of human papillomavirus (HPV) [3].

Various prophylactic HPV vaccines have been licenced for use in preventing HPV infection. The tetravalent vaccine combines HPV-16, -18, -6 and -11 virus-like particles (VLPs) to trigger an immune response that can prevent infection by these HPV types. A nonavalent version, has also been licenced to extend the protection against other oncogenic HPV types [4]. These vaccines reduce the burden of HPV-related cancers including cervical cancer, as well as anogenital and head and neck cancers [3]. They were originally licenced for adolescent girls before sexual debut but have been subsequently extended to boys as well [5, 6].

The vaccines are expected to produce nearly complete protection against HPV types responsible for over 70% of cervical cancers and their pre-neoplastic lesions, at least in the short term by a mechanism primarily mediated by neutralizing antibodies [7, 8]. However, one key limitation is that antibody levels elicited through vaccination may wane over time [9], therefore their long-term durability needs to be investigated.

Immunogenicity testing is important for a number of good reasons. Specific population-based evaluation of vaccine immunogenicity is significant for generating robust evidence of vaccine effectiveness and paving the way for the implementation of large-scale universal vaccination exercises. Immunogenicity studies may also help to clarify the anticipated duration of protection for vaccinated individuals and optimize universal vaccination protocols in the long-term [10].

Neutralization assays to estimate circulating anti-HPV antibody levels have been developed as the ‘gold standard’ for monitoring immune status during clinical trials or following the introduction of HPV vaccines. In addition, immunogenicity studies based on single epitope-based inhibition immunoassays and enzyme-linked immunoassays (ELISAs) have been reported [11–13]. Multiple-epitope neutralization assays are difficult to optimize and deploy in large scale clinical trials and implement in resource-constrained settings. However, ELISAs have the advantage of being sensitive, rapid, reproducible and amenable to automation [10]. ELISA technology is considered to be an excellent surrogate for neutralizing activity for evaluating antibody response induced by L1 VLP-based cervical cancer vaccines, regardless of time elapsed after vaccination (up to 6.4 years) and the age of the vaccine recipient [10].

Despite approval for use of the vaccine in some of African countries, data on immunogenicity and safety of HPV vaccines in sub-Saharan Africa remains scant [11, 12]. The GAVI HPV vaccine cohort presented an ideal opportunity to optimize and implement an indirect ELISA assay based on multiple epitopes, for the quantitation of HPV-16/18/6/11 antibody responses in vaccinated Ghanaian girls 36 months after the last vaccine dose.

## Methods

### Study setting and population

The Ministry of Health, Ghana, in collaboration with the Global Alliance for Vaccines and Immunization (GAVI) implemented HPV vaccination among in-school (class 4) adolescents in 4 selected districts; Shai Osudoku and Ningo Prampram in the Greater Accra Region, and Sagnarigu and Tamale in the Northern Region [14, 15]. The first phase of the project was implemented from November 2013 to May 2014. A total of 7,067 girls were vaccinated in this phase, with 6,770 (95.8%) of them receiving all three doses [16].

A retrospective cohort of in-school vaccinated pre-adolescent girls from the first phase of the GAVI HPV Vaccination Pilot Project in Ghana were age-matched with unvaccinated pre-adolescent girls in a control arm in a 1:1 ratio, from May to July 2017, 36 months after receiving the final dose of the HPV vaccine. Girls who received all 3 doses of the vaccine in the 4 districts were eligible for inclusion into the vaccinated group. Unvaccinated girls in the same class (as the vaccinated ones) from different schools in two neighbouring unvaccinated districts (one in each of the two regions) were selected for inclusion into the control group. Only girls who were still enrolled in the same schools at the time of the study were included. Girls who did not receive all three doses of the HPV vaccine during phase 1 of the GAVI pilot, those who received all three doses of HPV vaccine during phase 1 but had been repeated or had changed schools since the vaccination, and unvaccinated girls from vaccinated schools were excluded from the study. Written informed parental consent as well as assent from girls were obtained prior to enrolment.

#### Selection of districts, schools and girls

In each of the two regions, one unvaccinated district was selected as a control district; Ashaiman and Savelugu Districts in the Greater Accra and Northern regions respectively. Within each vaccinated district, 2–3 basic schools with the highest number of vaccinated pupils were selected. For the unvaccinated districts, two basic schools were selected from Ashaiman District and three basic schools from Savelugu District. The number of pupils selected from each school was proportional to the number of vaccinated pupils in the school (for vaccinated districts) and the number of pupils in the corresponding class (pupils in Junior High School Form One [JHS 1] in the 2016/2017 academic year) in the unvaccinated schools. Within each school, pupils in JHS 1 (those who were in class 4 in 2014) were systematically selected using the vaccination and class registers for vaccinated and unvaccinated schools respectively as the sampling frame. The sampling interval varied from one in two to one in four, depending on the number of eligible pupils, with the first being selected randomly.

Prior to sample collection, the study team visited each school to engage school authorities on how to conduct the study without disrupting regular school activities. The head teacher and the study team agreed on the appropriate dates and allocated time to conduct the study in each institution.

### Sample size estimation

Sample size calculations were done in Stata version 12 (StataCorp, College Station, Texas USA). Assuming that at least 50% of the girls that were vaccinated remain seropositive for HPV 6, 11, 16 and 18 antibodies after 3 years, and that not more than 30% of unvaccinated in-school girls in the community were seropositive for these antibodies, an estimated sample size of 304 girls (152 vaccinated and 152 unvaccinated girls) gave adequate power (90%) to detect these proportions. The number of girls selected from each vaccinated or unvaccinated district was in proportion to the size of the vaccinated cohort or unvaccinated pupils from the respective district. For the purposes of this proof-of-concept, 89 samples (from 49 vaccinated and 40 unvaccinated pupils) were selected for testing.

### Data collection

In all schools, head teachers invited parents/guardians of girls on agreed dates to explain study procedures and seek consent prior to school visits. The research team was invited to answer/clarify any outstanding issues before obtaining consent. On the day of the school visit, eligible pupils whose parents/guardians consented to their participation in the study were selected by systematic sampling. A member of the study team contacted selected girls individually and explained the purposes, benefits and procedures of the study and obtained written assent. Eligible pupils who were not available during the visit or who declined assent were excluded from the study and replaced with the next consecutive person on the register. Assenting pupils gave confidential interviews in private rooms.

### Sample collection and handling

After each interview, 5 ml of venous blood was collected from each participant into serum separator tubes (SST) by a phlebotomist/laboratory technician under standard aseptic conditions and allowed to clot at room temperature. Serum was aliquoted into sterile Eppendorf tubes in duplicate after centrifugation. Serum samples were then transported on ice to the Regional Public Health Reference Laboratory within 2 h of collection. All samples were subsequently transported to the Haematology Laboratory of the Greater Accra Regional Hospital for testing and determination of antibody titres.

### Serial dilution of samples and controls

In order to minimize non-specific binding, calibrator samples were prepared as five-fold serially diluted samples. Frozen serum samples were thawed and diluted 100-fold with a proprietary buffer consisting of protein stabilizer, detergents and anti-microbial agent. All assay runs were internally controlled using known positive and negative controls in duplicate. In addition, a set of calibrators, which serve as internal controls were employed in duplicate. The 1 U/ml low signal calibrator was used to discriminate the positive/negative threshold. All samples were run in quadruplicate to provide reliable serial dilution curves for accurate estimation of antibody titres.

### Serological evaluation of HPV antibodies

Samples from vaccinated girls and age-matched controls were tested using an indirect anti-HPV antibody ELISA kit (Alpha Diagnostics, USA) approved for research, according to the manufacturer’s instructions. In brief, quantitation of anti-VLP antibodies was performed using HPV-16 or -18 L1 VLP as coating antigen (2.1 μg/mL and 2.7 μg/mL, respectively). Diluted serum samples were added to VLP-coated plates and incubated for 1 h at room temperature while shaking [17]. Following a washing step, horse-radish peroxidase-conjugated anti-human IgG was added to each well as the secondary antibody and reactions were incubated for 30 min at room temperature, followed by incubation with tetramethylbenzidine (TMB) for a further 15 min [17]. Wells were treated with dilute sulphuric acid (0.18 M) to terminate reactions before optical density readings were taken at 450/620 nm. All laboratory staff were blinded to the study data throughout the laboratory analysis stage and could not tell which samples were obtained from vaccinated or unvaccinated individuals as samples were labelled with codes.

### Assay performance

The ELISA methodology developed permitted the qualitative classification of assay results as seropositive or seronegative status based on the optical density (OD) values returned by known low dilution positive calibrators. By an iteration of L1 antigen and HRP conjugate concentrations, the assay final was capable of discriminating anti-HPV IgG from background (non-antibody) signal from serum samples diluted 100-fold. In order to prevent false negatives, an alternative threshold, a positive index, could be established with a pool of confirmed negative serum or by using confirmed internal controls as discriminator (in place of the 1 U/ml calibrator control provided in the kit). After assay﻿ run, a strongly positive correlation was present for the calibrator series used and their OD values indicating assay sensitivity to increasing levels of antibody activity.

### Interpretation of results

A. Antibody activity threshold index

Antibody activity of serum samples was expressed as a threshold index obtained relative to the 1 U/ml calibrator. In this approach, the ability of the assay to report type-specific antibody activity, from either natural infection or vaccination, is dependent on the 1 U/ml calibrator which represents a threshold optical density of 1.0 for true positives at a 100-fold dilution or greater in a well-performed assay. OD readings above the 1.0 index are indicative of positive antibody activity while those below the 1.0 index can be considered negative for antibody activity.

B. Positivity index

Antibody activity of serum samples was expressed as a threshold index obtained in comparison to control or non-immune samples, by calculation of a positive index. This was calculated as the sum of the mean OD on one hand and the standard deviation (SD) of the control/non-immune samples multiplied by a factor of 2 (mean OD + 2SD). The antibody index was obtained by dividing each sample net OD reading by the positive index. Values above 1.0 were indicative of positive antibody activity and values below 1.0 were considered negative for antibody. This calculation classified samples with OD values clearly above the mean OD value of the pre-immune serum sample or a suitably determined non-immune panel or pool of samples tested at the same sample dilution, as positive and also estimates the antibody activity level [18].

#### Data analysis

The proportions of girls who were seropositive for anti-HPV 16 and 18 were calculated and compared among vaccinated and unvaccinated girls. Differences between proportions were evaluated by one-sided p-values for chi square test. Geometric mean antibody titres (GMATs) were calculated with 95% confidence intervals and compared between vaccinated and unvaccinated girls using the students’ t test. P < 0.05 was considered statistically significant.

## Results

### Demographic characteristics

A total of 89 samples were selected for testing; 49 from vaccinated and 40 from unvaccinated pupils. All the vaccinated samples were from Tamale Metropolis and the unvaccinated controls from Savelugu District. The demographic characteristics of the study population are shown in Table 1. The mean ages of the vaccinated and unvaccinated girls were not significantly different (15.1 vs 15.8; p = 0.116). Almost all the participants (97.9%) were of Mole-Dagbani lineage or Muslims.

**Table 1:** Demographic characteristics of study population

Parameter	Vaccinated n (%)	Unvaccinated n (%)	Total N (%)
**Age** (mean (SD))	15.2 (1.6)	15.8 (1.6)	15.5 (1.6)
**District**			
Tamale metro	49 (100.0)	0 (0.0)	49 (55.1)
Savelugu	0 (0.0)	40 (100.0)	40 (44.9)
Ethnicity*			
Mole-dagbani	46 (97.9)	40 (100.0)	87 (98.9)
Akan	1 (2.1)	0 (0.0)	1 (1.1)
Religion*			
Christian	1 (2.1)	0 (0.0)	1 (1.1)
Muslim	46 (97.9)	40 (100.0)	87 (98.9)

*Information on ethnicity and religion missing for two girls

### Prevalence of seropositivity

Seropositivity results, computed using the low calibrator (1U/ml) OD or a known blank well as a reference are reported in Table 2. Samples with OD greater than 1 were considered as positive and borderline values were defined for samples within ± 0.1 of the reference. For both HPV-16 (63.3% vs. 12.5%; p < 0.001) and HPV-18 (34.7% vs. 20.0%; p = 0.042), the seroprevalence of anti-HPV IgG antibodies was significantly higher for vaccinated girls compared to unvaccinated girls, respectively.

**Table 2:** Serostatus of vaccinated and unvaccinated girls

	Vaccine n = 49 n (%)	Control n = 40 n (%)	P-value
**HPV-16**			< 0.001
Positive	31 (63.3)	5 (12.5)	
Borderline	14 (28.6)	2 (5.0)	
**HPV-18**			0.042
Positive	17 (34.7)	8 (20.0)	
Borderline	5 (10.2)	2 (5.0)	

### Antibody responses: index method

**Fig. 1:**
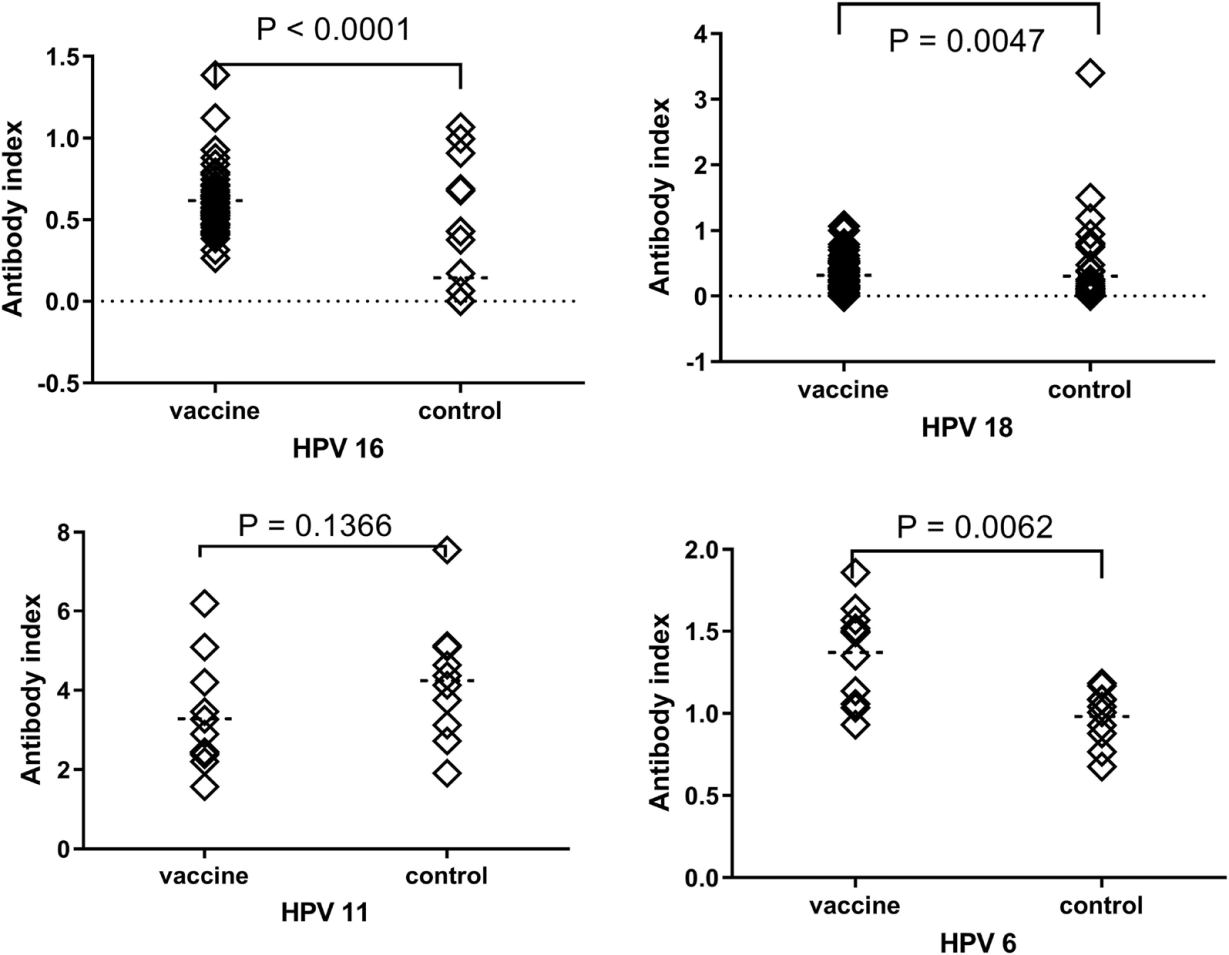
Mean antibody levels of vaccinated and unvaccinated girls (Method 1)

Antibody responses among vaccinated and unvaccinated girls according to the threshold antibody levels for vaccine-naive individuals (Method 1) are shown in Fig. 1. Among vaccinated girls, significantly higher mean anti-HPV-16 (0.618, 95% CI 0.559–0.677 vs. 0.145, 95% CI 0.048–0.242), anti-HPV-18 (0.323, 95% CI 0.250–0.396 vs. 0.309, 95% CI 0.111–0.507), and anti-HPV-6 (1.371, 95% CI 1.174–1.568 vs. 0.981, 95% CI 0.861–1.101) antibody levels were measured 36 months after receiving the third dose of vaccine compared to unvaccinated girls (p < 0.05).

### Antibody responses: threshold method

**Fig. 2:**
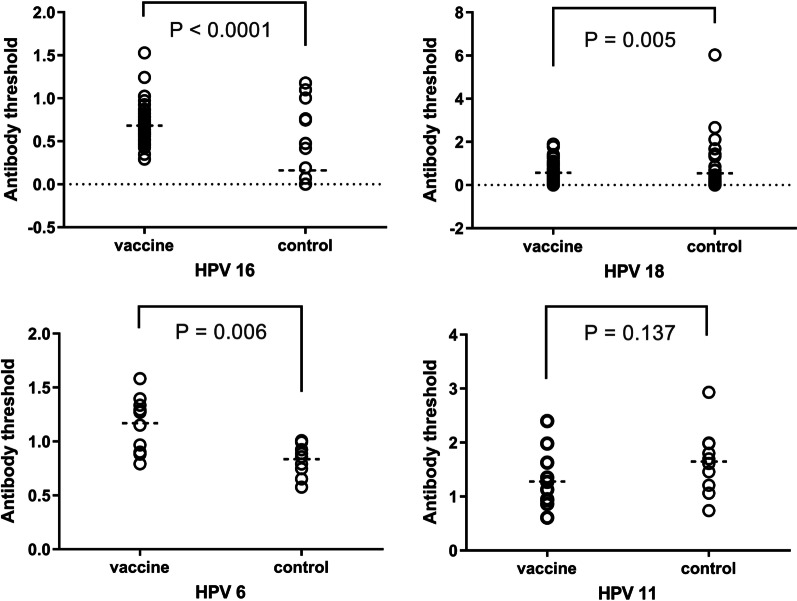
Mean antibody levels of vaccinated and unvaccinated girls (Method 2)

Antibody responses among vaccinated and unvaccinated girls indexed against a non-immune threshold sample (1U/ml calibrator) (Method 2) are shown in Fig. 2. Results for this approach were similar to those obtained for the Index Method. Among vaccinated girls, significantly higher mean anti-HPV-16 (0.682, 95% CI 0.617–0.748 vs. 0.160, 95% CI 0.053–0.268), anti-HPV-18 (0.573, 95% CI 0.443–0.703 vs. 0.548, 95% CI 0.197–0.899), and anti-HPV-6 (1.168, 95% CI 1.000–1.336 vs. 0.836, 95% CI 0.734–0.938) antibody levels were measured 36 months after receiving the third dose of vaccine compared to the comparison group (p < 0.05).

### Antibody responses: titer method

**Fig. 3:**
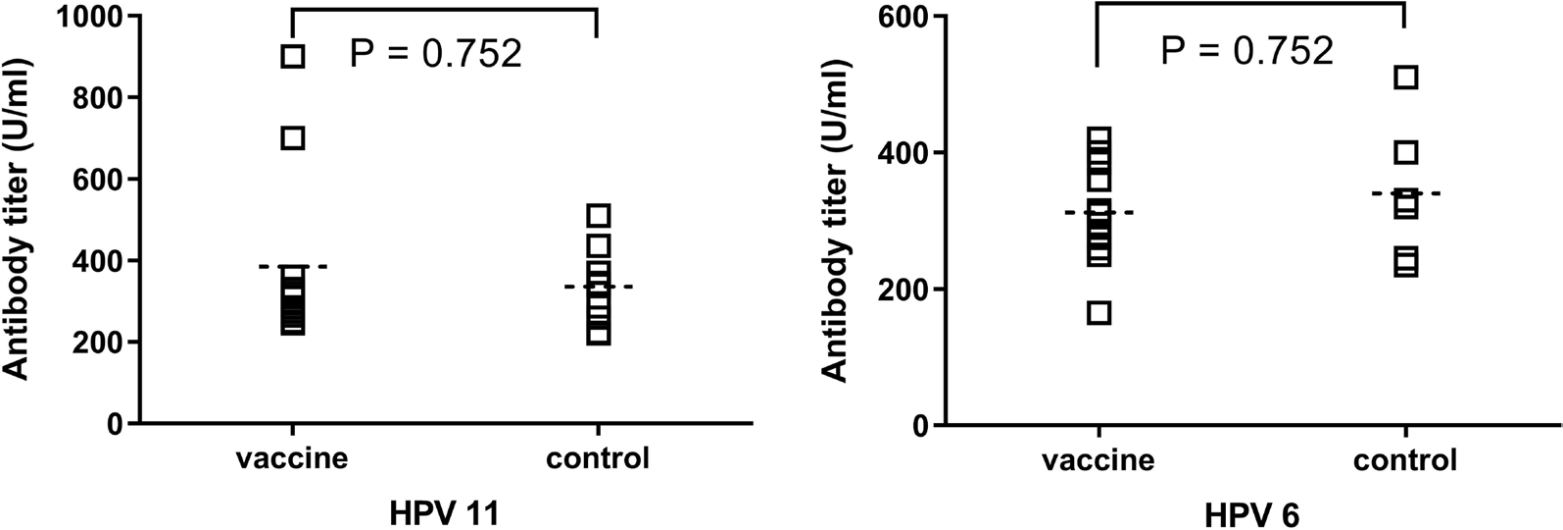
Mean antibody levels of vaccinated and unvaccinated girls (Method 3)

The antibody titers measured for vaccinated and unvaccinated girls (Method 3) are shown in Fig. 3. Among a small non-representative sample screened, the median antibody titer for vaccinated girls was not significantly different from that for unvaccinated girls for both HPV 6 (310.0, IQR = 260.0–385.0 vs. 325.0, IQR = 242.5–427.5) and HPV 11 (310.0, IQR = 265.0–360.0 vs. 324.5, IQR = 250.8–386.5). The GMATs for vaccinated girls versus unvaccinated girls were as follows for HPV 6 (349.3, 95% CI 262.3–451.1 vs. 324.9, 95% CI 266.7–395.9) and HPV 11 (303.0, 95% CI 253.3–362.6 vs. 327.8, 95% CI 240.8–446.1), respectively.

## Discussion

We have optimized and implemented a robust, scalable and sensitive assay for the evaluation of IgG antibody responses to vaccination with HPV-6, -11, -16 and -18 VLPs. The seroprevalence of HPV 16 and HPV 18 anti-HPV IgG antibodies was significantly higher among vaccinated than unvaccinated girls, as were the mean anti-HPV antibody levels. The median antibody titers did not differ significantly between vaccinated and unvaccinated girls.

Neutralizing antibodies are perceived to be the single most important determinant of prophylactic HPV vaccine performance [13]. As such, their long-term persistence in human serum is consistently a matter of significance in population-based vaccine studies [10]. A number of assays such as ELISA, competitive Luminex immunoassays (cLIA) and in-vitro neutralization assays are available for evaluating the viability of the immune response in clinical trials and in post-vaccination surveillance [8]. Among these, ELISAs stand out for being versatile enough to be automated on third-party laboratory systems to reduce cost, rapidly scalable, and can be optimized to quantitatively measure neutralizing antibodies as a marker of vaccine durability [10].

Antibody titres estimated from serial dilution curves of test samples provide accurate reflection of the strength of the immune response to antigenic challenge. Antibodies of the immunoglobulin G class were demonstrated to be within detectable limits within serum drawn from the GAVI/EPI immunization pilot 36 months prior. There was indication that this antibody activity was more pronounced in vaccinated girls compared to non-vaccinated HPV-naïve girls or in natural infection. Since the GAVI/EPI-sponsored pilot vaccination of pre-adolescent girls in primary 4 and 5 some years ago, the young girls will now be in their mid-late adolescence with likely initiation of sexual activity and heightened risk of HPV infection [5, 19].

The rates of seroconversion to HPV-18 and 16 at month 36 observed here are consistent with other studies [5, 19, 20]. Current seropositivity assays utilizing spectroscopy suffer from significant background noise especially at low concentration. This may cause loss of sensitivity at antibody levels typical of individuals vaccinated several years prior as well as inability to distinguish between low antibody titres from non-immune sera [20]. More sensitive antibody assays should make it possible to prove this hypothesis. Qualitative assays like this help to circumvent the problem of false negatives associated with qualitative assays by estimating an antibody titer rather than reporting a categorical outcome. In the meantime, the evidence from studies conducting repeat testing suggests that the prevalence of vaccine-type HPV infection among immunized girls is very low [21].

The antibody activity/titer detected in this work would suggest that the strength of the initial immune response may have waned according to the expected kinetics of anti-HPV antibody responses [13, 22]. Previous studies in HIV-negative girls and young women from sub-Saharan Africa indicate that the time-curve for antibody activity following vaccination with HPV VLPs follows a bell-shape with a peak antibody concentration expected approximately 4 months following the third dose of vaccine with sub-peak antibody titers detected 12 months after the initial dose [5, 23–26]. Some modeling results have predicted that the protection induced by currently available vaccines should last for at least 21 years irrespective of the vaccination schedule [13, 27]. Our results provide empirical evidence that the initial antibody response is sustained even at 36 months post vaccination and around the average age of sexual debut for African girls [28]. These findings should reinvigorate the discussion to introduce prophylactic HPV vaccination of girls as a national strategy for preventing HPV infection and its sequelae among women in the country. The persistence of these antibodies will be expected to confer much-needed protection against HPV genotypes covered by the vaccine [29]. Cross-protection to closely-related genotypes may also be expected [30, 31].

All the girls who received the vaccine were enrolled in school at the time of vaccination making it easy for follow up. However, it is unlikely that this may have significantly affected the outcome of this study. There is evidence to suggest that a similar outcome could be expected for girls who receive the vaccine in other settings provided they are able to receive all three doses [7, 22].

In most cases except with HPV-11-targeted antibodies, vaccinated girls registered a significantly increased antibody index/threshold and sero-conversion rate relative to unvaccinated girls. This could be as a result of a number of reasons. It is conceivable that the initial response had waned to natural levels or the small sample size of this pilot was inadequate to detect such significant differences. Ultimately, larger studies may be required to clarify this observation.

## Limitation of protocol

The assay used in this work is designed to recognize and quantify anti-L1 protein specific IgG antibodies. This will limit the performance of the assay in individuals with who, although infected, fail to produce L1 protein specific antibodies. Also, Anti-HPV antibody levels can be undetectable when below a minimum threshold titre when sampling is done too soon after infection. Detectable antibodies may also arise from samples taken from individuals with prior exposure and not necessarily from the vaccine. However, these limitations do not apply to vaccinated individuals and do not diminish the significance of the study’s findings.

## Conclusion

Our results indicate the presence of immunoglobulin G antibody activity within serum drawn from the GAVI/EPI immunization pilot 36 months prior. Anti-body detection was more pronounced in vaccinated girls compared to non-vaccinated HPV-naïve girls or in natural infection. We have successfully optimized and implemented a robust and sensitive assay for the evaluation of antibody responses among immunized adolescent girls. This protocol will be useful for future large-scale HPV vaccination studies in low-income settings.

## Supplementary Information

**Additional file 1: S1 File:** Demographic Data.

**Additional file 2: S2 File:** Seropositivity data.

**Additional file 3: S3 File:** Antibody data.

## Data Availability

All data generated or analysed during this study are included in this published article and its supplementary information files (Additional file 1: S1 File: Demographic data, Additional file 2: S2 File: Seropositivity data, Additional file 3: S3 File: Antibody data).

## Abbreviations

L1: Late viral capsid protein
IgG: Immunoglobulin
GAVI: Global Alliance for Vaccines Initiative
HPV: Human papillomavirus
CHRPE: Committee for human research publication and ethics
Anti-HPV: Antibodies directed against human papillomavirus
ELISA: Enzyme-linked immunosorbent assay
OD: Optical density
GHS: Ghana health service
EPI: Extended Program on Immunization
KNUST: Kwame Nkrumah University of Science and Technology
GSK: Glaxo Smith Kline
IQR: Interquartile range
VLP: Virus-like particle
